# Determining priorities for research to improve fundamental care on hospital wards

**DOI:** 10.1186/s40900-016-0045-8

**Published:** 2016-10-12

**Authors:** Jane Ball, Claire Ballinger, Anya De Iongh, Chiara Dall’Ora, Sally Crowe, Peter Griffiths

**Affiliations:** 1 National Institute for Health Research Collaboration for Leadership in Applied Health Research and Care (CLAHRC), Wessex, UK; 2 University of Southampton, Building 67, Highfield Campus, Southampton, SO17 1BJ UK; 3 Doctoral Student, Karolinska Insitutet, Stockholm, Sweden; 4 Crowe Associates, Thame, UK

**Keywords:** Priority setting, Fundamental care, Hospital wards, Nursing, Public involvement

## Abstract

**Plain English summary:**

The aim of this project was to find out the priorities for research that could improve fundamental care. ‘Fundamental care’ covers all aspects of basic care in hospital wards, such as helping with core physical needs, building positive relationships and keeping patients safe.

By setting the priorities with patients, carers, the public and health care professionals, research can look at the issues that really matter to people who are receiving or delivering care in hospital wards.

Previously, prioritisation exercises have started with a menu of options and asked people to choose from that list. They have also been focused on specific health conditions. Traditionally, there has been little opportunity for patients, carers and the public to contribute to identifying the issues to be prioritised.

To develop the priorities for research, we started by exploring what is meant by ‘fundamental care’, looking at patient and carer accounts and academic and policy reports. Patients, carers, staff, and members of the public were consulted via surveys, interviews and group discussions to share experiences and issues.

A list of 15 topics was identified based on what was most commonly mentioned by patients, carers and healthcare professionals as well as what was practical for the CLAHRC Wessex team to research. A workshop with patients, carers and healthcare professionals was held, to decide the top 5 areas.

The five priority areas identified were:

- Nurse staffing
- Individualised patient care
- Staff communication
- Staff attitudes and relationships with patients
- Information about care/communication

**Abstract:**

**Background**

The provision of high quality fundamental care in hospitals is a top priority for the NHS. Recent reports and investigations highlight that at times care has fallen below standard. It is unclear what research should be prioritised to improve care. The aim of this work is to involve patients/carers/public, clinicians and other stakeholders to identify issues that are priorities for research which could improve fundamental care in hospital.

**Methods**

Patient and public involvement was integral to this project, with a patient leader/service user being a member of the core team who designed and executed this research. After consideration of existing priority setting approaches, we developed an inclusive approach which consisted of six main phases: 1) Development of a conceptual framework of fundamental care, based on reports and literature 2) Consultation with a wide range of stakeholders through a survey, focus groups and interviews 3) Identifying themes from the responses to the consultation phase (76 themes identified) 4) Analysis to identify the 15 topics most frequently cited 5) Prioritisation of the top 15 themes through a half day workshop, which led to a shortlist of five themes 6) Development of the top 5 themes into research areas.

**Results**

Three hundred forty stakeholders (29 % of whom were patients/carers/public) completed the consultation survey. Analysis of the survey responses and of focus groups and interviews led us to identify 15 high scoring themes. We presented these at the prioritisation workshop, attended by 39 participants (23 of whom patients/carers/public). After a voting exercise, the 5 top research priorities which emerged were: nurse staffing; individualised patient care; staff communication; staff attitudes and relationships with patients; and information about care.

**Conclusions**

We involved a range of stakeholders in identifying topics for research to improve fundamental care and asked them to prioritise these. The process provided a means of reaching consensus as to the important issues for future research to focus on to improve fundamental care on hospital wards.

## Background

Despite the commitment to quality of care set out in the NHS Constitution [1], fundamental care is not always provided to the high standards that patients have a right to expect. A series of investigations into high profile failures and numerous reports [2, 3] have highlighted substantial and significant variations in the quality of fundamental care provided to patients in NHS hospitals [4–6]. Training, staffing levels, leadership, motivation and organisational culture are all implicated in failures of fundamental care [7, 8].

A number of initiatives have been proposed to improve the capacity of hospital nurses to deliver safe and effective fundamental care. These range from single interventions such as guidelines on specific aspects of care, through to organisational initiatives such as routine reporting of adverse events (e.g. the safety thermometer [9]), or quality improvement programmes such as the “productive ward” [10]. However, research to demonstrate the impact of these initiatives is often lacking. Few studies of nursing interventions to improve fundamental care use robust methodologies that allow the results to be applied to other contexts and there is little evidence of a programmatic approach [11].

Fundamental care on acute hospital wards can refer to any element of the nursing care or the factors that influence the delivery of that care. It covers a wide range of elements, and can be viewed from the perspective of a patient receiving care, staff providing it, the systems/procedures involved in delivery, or the net effect of that care in terms of outcomes. It is thus a broad term that may be subject to differences in interpretation. Kitson and colleagues set out to define fundamental care drawing on nursing literature, arguing that such a synthesis is needed to improve care delivery by improving patient safety and quality initiatives [12]. Marshall and colleagues highlight the importance of involving patients in defining and conceptualising our notions of what constitutes patient centred care [13].

In this paper we report on work to determine priorities for research to improve fundamental care as part of the work of the National Institute for Health Research Collaboration for Leadership in Applied Health Research and Care Wessex (NIHR CLAHRC Wessex). We proposed three areas at the outset: activities designed to meet core physical needs (such as eating and drinking, elimination and continence, and skin care), establishing positive relationships (e.g. treating patients with respect), and maintaining patient safety. Our idea of what constituted fundamental care thus drew upon elements from the ‘activities of daily living’, whilst also encompassing the way in which care is delivered-interactions, not just transactions, and a principle of nursing espoused by Florence Nightingale-that the ‘hospital should do the patient no harm’ [14].

In the face of the multiple, complex issues and wide range of possible solutions, combined with limited capacity to study every issue, prioritisation involving all stakeholders was needed to determine which areas of research have the greatest potential to improve practice and benefit patients. Hence our endeavour to involve others-patients, their carers/families, members of the public, clinicians, managers and commissioners-in determining topical issues and priorities for research to improve fundamental care.

### Determining priorities for Research

For as long as health research has been undertaken, there have been factors that influence the choice of what it is that is studied. In the 1990s, research in the NHS was criticised for being conducted in a ‘piecemeal fashion’ without strategy or clear leadership [15]. To remedy this, priority-setting activities started to be undertaken. This typically involved groups of ‘experts’-normally academics-being convened to identify research gaps to inform funding decisions. The public and patient voice was absent from these discussions and perhaps as a result it was found that research and development funding in the NHS was not focused where it was needed but was locked into “historical allocations” [15, 16].

The aim of establishing the ‘National Institute for Health Research’ (NIHR) was to improve coordination in health research funding, and ensure that research findings could be applied to health services, and the patients and public they serve. The NIHR (in common with other bodies) has been proactive in considering how to prioritise the research topics they fund, and involving patients and the public in this endeavour.

The James Lind Alliance (JLA) was established in 2004. The JLA developed an approach involving both health care professionals and patients, known as ‘Priority Setting Partnerships’ [17]. Each Priority Setting Partnership is regarded as potentially different, but all are based on a set of common principles, and typically involve a three stage process: a survey; an interim priority exercise (online); and a final prioritisation exercise (face to face). The core principles are: transparency of the process, balanced inclusion of patients and clinicians, exclusion of non-clinician researchers for voting purposes, exclusion of groups with significant competing interests (e.g. pharmaceutical companies), audit trail of the research topics through to final prioritised list, and recognition that priority setting does not create new knowledge-it is a form of shared decision making, not research [18]. Between 2007 and 2014 the JLA approach has been used to guide more than forty priority setting partnerships. However, a comparison between research priorities identified and research being funded identified a mismatch between the different stakeholders in the process [19].

Although there is broad agreement that research priority setting processes can help target research and enable greater relevance, there is no clear view regarding the best way of conducting them [20]. Indeed the way in which priority-setting is done, and the methods used, can influence the outcomes of the process [21].

We developed an approach to prioritisation that builds on the strengths of previously used methods, and which gives a mix of stakeholders the opportunity to identify and define the topics, as well as prioritising them. We sought to engage with staff and patients/carers/public to consider how the short-listed topics could be developed into research, and get stakeholder views on the criteria by which future research should be judged. Such a strategy-that goes beyond prioritising topics initially identified by researchers-was considered to be particularly important in identifying research topics and priorities in what is a broad area. Many priority-setting exercises have been applied to specific treatment groups, rather than large and less tightly defined areas such as ‘fundamental care’.

We wanted an approach that allowed stakeholders to elucidate as well as prioritise the topics for research. Using a food analogy, to give ‘consumers’ not just a choice from the menu, but control over what is on the menu, the type of food or the style of cooking.

Priority setting is typically undertaken on behalf of a funding body, to decide how to target research funds. Once priorities have been identified, they are translated into research specifications and researchers invited to put forward proposals. However, in our context, the funding had already been allocated to NIHR CLAHRC Wessex, and it was the research team, not the funder that was seeking to prioritise future research within the ‘Fundamental Care’ theme. A different type of partnership was required to maximise the input of stakeholders outside the research team, whilst pragmatically working within resource constraints.

## Aim

The aim of the project was to determine areas for research to improve fundamental care on hospital wards by developing and applying an inclusive approach to research prioritisation.

## Methods

The project team comprised two researchers, an experienced patient leader (who is a service user) and a Patient and Public Involvement lead. The Term ‘patient leader’, refers to a patient/service user who works with, and for others, to influence decision-making at a strategic level [22]. The inclusion of both a patient leader and a PPI lead was key to the development of an approach and the design of the project, to ensure all aspects of the process were considered from a patient and public perspective. No decisions about the process were taken without the involvement and agreement of the whole team.

**Fig. 1:**
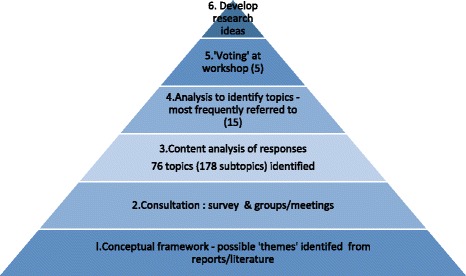
The six phases in the priority setting process

After an initial review of priority setting approaches (both through the literature and meeting with people involved in similar priority setting exercises), we set about developing an inclusive approach that would be suitable to determine areas for research to improve fundamental care on hospital wards. The approach developed consisted of six main phases (see Fig. 1).

The perspectives identified in phase 1 (conceptual framework) were used to inform the design of the second phase (consultation survey). We returned to our broad framework of fundamental care to help identify themes emerging from the consultation survey and structure the analysis of the responses to the consultation (in phase 3). We analysed the responses quantitatively to identify the prevalence and relative priority of themes, to generate a long list of themes (phase 4). At a workshop for staff, patients and members of the public we used voting techniques to produce a shortlist of five topics (phase 5), which were developed into potential research topics through small mixed groups of stakeholders (phase 6).

1. Identifying and defining terms: what do we mean by ‘fundamental care’?
  Drawing on academic literature, practice guidelines, policies and reports, we started by outlining a theoretical framework for fundamental care, exploring how it is defined and identifying the types of topics encompassed, from the perspectives of patients, staff, academics and organisations (see Table 1).

**Table 1:** Sources drawn on in scoping ‘Fundamental Care’

		Number of topics identified
Activities of daily living	[26]	6
‘Essence of Care’	[27]	12
‘National survey of patients in hospitals’	[5]	15
‘Patient Stories 2013: Time to Change’	[4]	4
‘What matters to patients’	[28]	20
‘What matters to staff in the NHS’	[29]	4
Factors affecting quality: themes in research	[30, 31]	13
  The purpose of this phase was to start to build a conceptual framework: identifying the range of activities and perspectives that shape what is covered by the term ‘fundamental care’. This was used to underpin the design of the consultation survey (phase 2) and was revisited in developing a coding frame to analyse the responses (phase 3).
  It was evident from this exploration that different ways of looking at ‘fundamental care’-as an activity, a process, an experience, or a metric-are at work in shaping how we think about care, and the language used to capture it (see Table 2).

**Table 2:** Perspectives on terminology and conceptualisation of care (taking ‘drinking/hydration’ as an example)

	Who’s perspective?	
	Care giver	Care recipient
Goals/Objectives of care: activities of daily living that patients may require help with whilst in hospital	Maintain hydration	Have enough to drink
Action: the types of intervention undertaken by care providers (primarily nursing staff)	Assist with drinks, administer IV fluids	Be given drinks
Associated activity/resource to enable care need met	• Fluid balance charts/systems in place • Organisation of responsibilities between staff (roles) • Sufficient staff to ensure drinking assistance and fluid monitoring undertaken • Routines-water jugs provided, drinks rounds, drinks placed in reach, suitable drinking aids	• Nurses know what I’ve drunk • I know how to get drinks • Range/choice of drinks available • I’m given help when I need it to ensure I have enough drinks or other fluids • I have sufficient access to drinks
Consequences/Outcomes	Clinical outcomes/measures of successful hydration (and dehydration) - Fluid in-balance - Skin condition Evidence/outcomes of poor hydration - Increased risk of urinary tract infection - Impaired cognitive function	Experience associated with hydration or dehydration: - Thirst quenched/feel thirsty - Comfort/pleasure eg. ‘enjoying nice cup of tea’
  Taking hydration/drinking as an example, terminology in use around this function reflects different perspectives, which stem from two dimensions:
  Who-perspective of provider (staff) or recipient of care (patient), and
  When-position along the timeline-from abstract goal (hydration), through to actions/activity (providing drinks) and their consequences (no dehydration, reduced risk of urinary tract infection).
  We started to generate a model that encapsulated these elements, to provide a framework for thinking about fundamental care that could inform the rest of the priority setting project.
  Fundamental care can be thought of as the set of actions and interactions that happen at the point of care delivery. What these actions are, depends on the specific needs of patients, as understood by staff, and by the application of knowledge and skills to meet these needs. The range of activities encompassed thus depends in part on the mix of patients, but also on the staff available to meet needs. *How* these activities are done, and the nature of the interaction at the point of care, will also be shaped by factors related to both the individual member of staff, and the organisational context within which they are working. Two main sets of topics for the consultation were thus identified: activity to meet patient needs; and ward/contextual features that support the delivery of care.
2. Consultation
  The goal of the consultation phase was to seek feedback from a diverse mix of people, from across the Wessex region. To achieve this we collected data through a consultation survey (open to anyone to complete), focus groups and interviews. We asked people what they saw as the main issues in ‘fundamental care’, for examples of good and poor care (and what differentiates the two), and about what issues should be prioritised for research to help improve care on hospital wards.
  In order to ensure representation of diverse patient populations in the prioritisation process, the Patient Leader (also a service user) and PPI lead generated a matrix identifying potential contacts across the three geographic areas covered by CLAHRC Wessex, for the following groups: minority ethnic groups (including asylum seekers and refugees), frail elderly people, people with sensory and physical disabilities, people living with long term conditions, people with learning disabilities, and people with dementia. People from these groups in at least one area were invited to take part in the consultation either through the survey (online or paper-see below) or if this was not feasible (for example, for people with dementia), visits were arranged in order for the Patient Leader and PPI Lead to discuss recent hospital experiences and priorities in an accessible way. Examples of this latter format included questions about recent hospital experiences, what proved worrying or frustrating, and the identification of one object which would have improved their hospital experience. Detailed notes capturing these discussions were completed by the Patient Leader and PPI lead, and included in analysis (along with the results from the survey).
  - Survey
    The first questions of the survey (entitled ‘How can we improve care on hospital wards?’) asked people to indicate to what extent (on a scale of 1 to 5) they considered topics to be a priority for research. The topics presented were based on the scoping in phase 1, and included a list of 14 care activities (such as skin care, toileting, eating and drinking) and a second list of 13 factors that could be regarded as supporting the delivery of fundamental care (such as team work, equipment, and nurse staffing levels ie. the numbers of nursing staff on duty). Space was provided to suggest other topic areas for research.
    The second half of the survey concerned situations when care had gone well or had not gone well; respondents were asked to describe in their own words the situation and why they thought it had gone well (or badly). Finally we asked people how they thought we could tell if a hospital was getting fundamental care right for patients, to elucidate what people saw as meaningful indicators of success or failure.
    The survey was launched in May and closed in September 2015. The online survey was available on the NIHR CLAHRC Wessex website [23] and the link distributed through social media (Twitter) and by email to groups such as: Macmillan Voices, ‘My Health My Way’, local associations for visually impaired people, Youth Association, Race Equality Councils, Healthwatch, an Ageing Network, Patient Councils, and a support group for migrant workers.
  - Groups/interviews
    Face to face consultation was undertaken with 97 members of the public, patients, carers and a mix of staff, through meetings and small discussion groups. To minimise the burden on participants and maximise engagement, all meetings and discussion groups were undertaken ‘in-situ’ i.e. we visited participants in the places where they were congregating rather than asking them to come to us.
3. Identifying themes
  The responses to the consultation phase were content analysed to identify emerging themes, and develop a coding frame [24]. The coding frame built on the conceptual map that we had started to develop at the outset of the project, which we developed further and is depicted in Fig. 2.

**Fig. 2:**
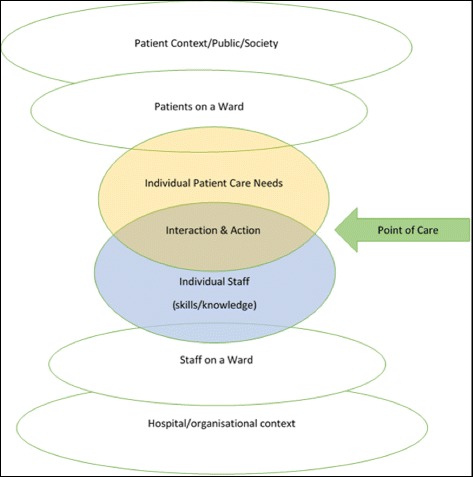
Conceptual framework for fundamental care: actions, interactions and context
  The coding frame identified 76 emerging themes, with a total of 178 sub-topics. The coding frame was shared with the entire project team-researchers and PPI-for review and validation. A first draft was used by 3 researchers to code a sample of data, and then the coding discussed to see if any further categories were required, and identify ambiguity. A revised ‘final’ coding frame was then produced. Two researchers coded the same sample of data independently to test inter-rater reliability and achieved matched codes on 93 % of items. A single researcher then coded all the responses. Any queries or ambiguities were raised with the senior researcher at regular intervals, and resolved by mutual agreement.
  All open-ended data from the consultation survey and the consultation groups/interviews-was subjected to this coding process, so that the frequency of themes could be quantified, and differences between subgroups (e.g. PPI and staff) within the survey could be examined.
4. Quantifying the most frequently cited themes (a ‘longlist’)
  We used descriptive statistics (counts and frequencies, cross-tabulations) to identify the most frequently recurring themes, and identify which were most highly prioritised-overall and by patient/public respondents in contrast to staff respondents. The results of this quantitative analysis were reviewed and the ‘top’ themes that were most highly cited in relation to different dimensions of enquiry/sources were flagged as being potential ‘top priority’ areas, thereby creating a list of 39 topics. The criteria used for inclusion into this list of top themes depended upon the data themselves; we looked for natural cut-off points that differentiated the responses to the themes. For example, a series of questions asked respondents to rate the priority that should be given to each of 29 topics on a scale from 1 (least) to 5 (most). Topics that scored an average of 4 or above for both staff and patients/public were selected for inclusion into the ‘long-list’. To compress the list further and reach a more manageable number of topics to present at the workshop, the research team reviewed the list of 39. Only topics that met at least two different sets of criteria were taken forward to the next phase (prioritising at the workshop). This reduced the longlist from 39 to 15 research areas.
5. Developing the top 5 themes into research topics
  We held a half-day stakeholder workshop (with 39 participants-23 PPI and 16 staff) to identify the relative priority of the 15 shortlisted topics, and considered the emerging top 5 research areas in more detail through small group discussions. The 15 priorities were mapped onto the conceptual framework, to help in presenting them to stakeholders for discussion (Fig. 3).

**Fig. 3:**
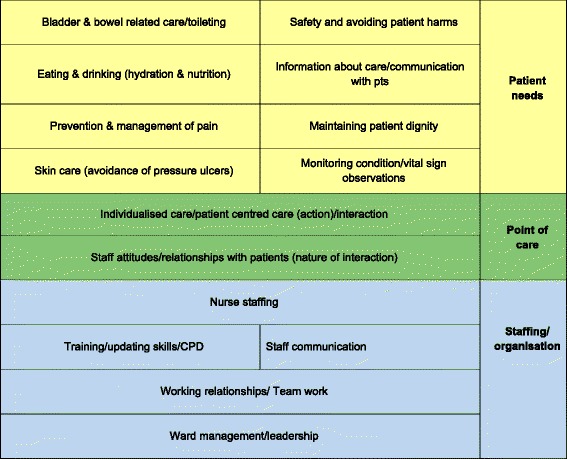
Categorisation of 15 priorities for Fundamental Care
  Everyone who had been involved in the consultation groups/interviews or had taken part in the consultation survey (who indicated their interest and willingness to get involved in further work and gave contact details) was invited. Our goal was to have a maximum of 40 participants-to allow plenary discussion and small group work-and to have a 50:50 mix of staff and patients/public, although in reality these terms were not mutually exclusive. Many participants wore a ‘variety of hats’, having experience of hospital as staff, patients, carers, and researchers. However, we used their own self-categorisation (in response to the question: Which best describes you?) as the basis of our grouping, both for the workshop and the analysis of the consultation responses.
  The four members of the project team, including the Patient Leader-Service User, were also involved, and acted as table coordinators/facilitators. The event was facilitated by an external consultant (with no vested interest in the outcome of the workshop) with considerable experience of priority setting, who worked with the project team to plan the event, consider the prioritisation methods, and develop workshop materials. Four other members of university staff helped with coordination and note-taking.
  The shortlisted topics were displayed as 15 A3 posters along one wall (with bulleted outlines of the sub-themes corresponding to each). Copies of information about each theme, including how it performed in the consultation survey, were also presented in the individual participant packs, and on the table. The facilitator introduced the 15 topics and invited the small table groups to spend some time making introductions and sharing their impressions of the current shortlist.
  A plenary discussion was facilitated to identify what participants saw as criteria against which to judge the topics that might influence their voting behaviour. Issues such as value for money, a wide application of the research outcomes and recognising the pressing need of older people in hospital were voiced, amongst others.
  Participants (excluding members of the research team and facilitators) then voted on the themes using a Nominal Group Technique [25]. Each participant had 10 dots to ‘spend’ on the exercise. They were encouraged to allocate: 3 dots once to allocate to their most important topic, 2 dots once for their second most important topic, and each of the remaining five dots could be distributed singularly elsewhere. Once all dots were used, the final scores were tallied. All topics with more than 20 dots were left at the top of the display boards for further consideration and those with less than 20 dots were relegated to the lower part of the display boards. Six themes were identified as top priorities, with four themes receiving scores of over 30.
6. Prioritising the top 15 themes through a half day workshop
  After discussion between workshop participants, the six highest-ranking topics were considered in small groups, for future research. Two of the six were integrated to form one theme, due to similarities in content, thus five topics were available for discussion. Each topic was allocated to a table (with average of 8 participants on each) to consider how the topic could be developed into a research question. Table groups discussed the topic, facilitated by NIHR CLAHRC Wessex staff who had a prompt question sheet to use to capture key information. They identified and discussed specific research questions within the topic area, and the type or design of research that might address these questions. At the end of the workshop each group presented their key ideas and discussion points to the wider group using flipcharts.

## Results

The first phase elucidated a range of perspectives on fundamental care and ways of categorising components of care, which was used to inform the approaches to priority setting and revisited as part of the analysis of responses and feedback.

A total of 340 people completed the consultation survey. Respondents comprised: members of the public, patients, and carers (29 % of respondents), registered nurses (30 %), other staff (22 %), students (7 %) educators/researchers (6 %) and others (6 %). The vast majority had experience (as patient, visitor, or staff) of one of the eight hospitals within Wessex. In 90 % of cases, that experience was within the last 3 years (71 % reported it was within the last 6 months).

Of the 29 topics presented, the themes that were ranked highest by both patients and staff (and the proportion of all respondents citing them) were: staff communication (89 %), nurse staffing levels (88 %), teamwork (84 %), eating and drinking (84 %), being able to communicate (84 %), safety and avoiding harm (79 %), and treating patients with dignity (77 %).

Analysis of the free text responses, and responses in the discussion groups, highlighted several ‘new’ issues, beyond those listed, that patients and staff felt were important to the delivery of fundamental care. Of these, three factors were viewed as key to determining whether care has gone well or not gone well: care that is individualised to the patient (including ‘patient centred care’), staff attitudes/relationships to patients, and communication about care. Just under half (49 %) of those describing factors that contributed to poor care referred to staff communication, with 31 % referring to staff attitudes and 28 % referring to staffing/workloads. Individualised patient care (also referred to as ‘patient centred care’) was referred to a factor in care that had gone well by 32 % of respondents, with 30 % citing good information given about care.

When asked “If there was just one topic that we could focus research on to improve fundamental care on hospital wards, what should it be?”, 30 % said nurse staffing levels, 17 % staff communication, 14 % patient dignity, 13 % ward management, and 12 % prevention and management of pain.

The themes that came out as high scoring on our multiple ‘tests’ were selected. Table 3 describes the resultant ‘long-list’ of 15 themes, how they were arrived at, and the rank position of each in the final voting exercise.

**Table 3:** ‘Longlist’ of 15 themes, how selected, & rank position in prioritising exercise

Rank position		High priority score (of listed topics in survey)	Often referred to as issue in GOOD care	Often referred to as issue in POOR care	Patient/public discussion groups	Staff discussion groups/interviews	If you had to pick just one topic	Researchers capability/expertise	Number of votes/score at the workshop
1	Nurse staffing levels/workloads	√	√	√		√	√	√	36
2=	Individualised care/patient centred care		√	√	√	√		√	34
2=	Staff communication	√	√	√	√	√	√	√	34
4	Staff attitudes/relationships with patients		√	√	√			√	31
5	Communication/Information about care		√		√				23
6	Ward management/leadership					√	√		22
7	Eating & drinking (hydration & nutrition)	√			√	√	√	√	21
8	Working relationships/Team work	√	√				√		18
9	Training/updating skills	√			√		√		17
10	Safety and avoiding patient harms	√					√		15
11	Maintaining patient dignity	√					√		12
12	Monitoring condition/observations	√				√		√	10
13=	Bladder & bowel related care						√	√	9
13=	Prevention & management of pain	√				√	√		9
15	Skin care (avoidance of pressure ulcers)	√				√		√	4

Following the voting activity at the workshop, five top priorities for future work to improve fundamental care on hospital wards were identified from the long-list of 15. A description of each with an illustrative quote is presented in Table 4.

**Table 4:** Five research priorities to improve fundamental care

1. Nurse Staffing (‘having time to care’, completing care, being able to respond to patient needs promptly, manageable workloads, not needing temporary staffing) “*Patients including myself had been buzzing for pain relief for over an hour*. (*kept being told* “*be there in a minute*”) […] *Waited nearly 2 h for pain meds. Nurses were tired*, *not enough staff*, *too many patients to staff ratio*”.
2. Individualised patient care (assessing care needs fully, care plans that take account of the individual, appropriate clinical care, patient centred care, ‘putting the patient first’, ‘treating the person not the condition’) “*my frail older mother was treated with respect and listened to*, *time was taken to ensure that she was treated as an individual and her past life* […] *It went well because my mother was treated as an individual*, *and was cared for with respect*”
3. Information about care and involvement (providing patients with information about their care, informed consent, enabling dialogue between patients and staff ‘hearing the patient’) “*From the start I was kept informed about what the process was*, *what was going to happen before during and after the operation. The experience was good because they managed the difficult balance of knowing absolutely what they were doing because they do it every day with the understanding that for me personally it was something happening just to me*”
4. Staff communication (communication between health care professionals, styles of communication, sharing information between staff/handover) “*Staff took the time to handover patients concerns to the next shift. Consistency of care was kept even though the staff changed*”
5. Staff attitudes/relationships with patients (staff relationships with patients, ethos and values, maintaining compassion, patients feeling staff ‘care’/they matter) “*Staff treated older patient as though she was nothing more than a nuisance and was getting on their nerves. Very distressing*.”

Table 5 describes the results from the consultation survey in relation to the top 5 themes.

**Table 5:** Priorities for research to improve fundamental care on hospital wards

Research themes (rank position after voting)	Views from the consultation
1. Nurse staffing	
- Having time (eg. member of staff taking time to find carer, time to do complete care) - Lack of time (eg. to respond to call bells, care undone) - Nurse workloads - Sufficient time for staff to be able to take breaks - Improving staffing levels - Use of agency staff (to cover shortages)	• When asked about care that had not gone well−28 % of survey respondents referred to staffing levels • Most frequent response to the question ‘if we could focus on just one factor care which would it be?’ (30 % selected it-twice as many as for other topics. It ranked ranking it no.1 out of 14) • Staff were more likely (37 %) than patients/public (14 %) to refer to ‘Nurse staffing’ as a priority topic
2 = Individualised patient care	
- Assessing patient care needs (fully) - Individualised care plans to meet patient needs (less reliance on standardised protocols) - Appropriate clinical care - Holistic/patient centred care - ‘Putting the patient first’ - Clear care plans - Treating the person not the condition	• This wasn’t listed as one of the ‘pre-set’ topics, but came up frequently in people’s answers on good and bad care, and what differentiates the two • Staff & patients/public both see this as key issue in good care (referred to by 32 % in the survey) • It emerges as an ‘over-arching theme’ to which all of the other topics connect
2 = Staff Communication (generally)	
- Between health care professionals - Sharing information between staff - Patient education: what to expect & how to have a say in their care - Style of communication-patient, skilful - Staff listening to patients - Honesty in communication	• Came up as a key issue in both staff and patient discussion groups • It was the most frequently discussed issue in relation to both good (43 %) and poor care (49 %)-and is the single issue accounting for most responses. • High ranking topic in response to ‘if we could only focus one topic’ (13 %) and had highest average research priority score (4.4 out of 5 for patients/public, 4.5 for staff) • Large proportion of patients/public refer to staff communication as factor when care has not gone well (60 %, vs 45 % staff)
4. Staff attitudes & relationships with patients	
- Ethos and values - ‘Care’ about patients - See patients as people - Maintaining compassion in staff - Relational care - ‘defensive medicine’ (making decisions on basis of being able to ‘defend’ them, rather than judgement of what is best for patient)	• An issue that came up as a theme in the patient/public discussion groups (closely linked to the communication themes) • The second most frequently cited issue raised in relation to quality of care-33 % refer to it in when talking about care that’s gone well, 31 % in relation to poor care.
5. Information about care/communication	
- Between staff and patients/carers - From patients to staff - Provision of information to patients - Informed consent procedures - Information (eg. using prompt sheet)	• One of the 3 topics that got highest ‘priority’ score (for both patients and staff-scoring 4.3 out of 5) • Some overlap with staff communication ‘generally’

## Discussion

With PPI embedded throughout the project through the inclusion of the Patient Leader-Service User as one of four members of the core team, and the involvement of patients, members of the public and staff, we identified five priority areas for research to improve fundamental care on hospital wards: nurse staffing, patient centred care, involvement in care, communication, and staff attitudes. The priorities have emerged from a ‘bottom-up’ approach that did not pre-empt the range of issues that stakeholders may have seen as important to the delivery of fundamental care. The emergent priorities for research point to a view of fundamental care that requires us to consider how different themes relate to one another, and build research that can address issues in tandem with one another, rather than focussing on single specific care activities.

The priorities for research are less focussed on specific care activities but relate more to underlying factors that contribute to the effective delivery of care (e.g. staffing and communication), as well as factors connected to *how* care is provided across all the ‘activities’ of care (individualised/patient-centred, tailored to meet the needs of the individual). This supports the view from a review of the literature, that whilst different stakeholders may emphasise different aspects of the ingredients of fundamental care, core themes relating to patient involvement, relationships, and the context of care can be identified [26].

The end-result of the process has been an insight into the issues that staff, patients, public and researchers consider important for future research to address, in order to improve fundamental care.

Several features of our approach helped us to give a broad cross-section of people the opportunity to describe the issues that they saw as important in the delivery of fundamental care. From the outset, the inclusion of the Patient Leader-Service User as a member of the core team ensured that our approach and methods were designed to reach patients with a wide variety of experiences and conditions. In the open consultation, as well as asking specifically about research priorities, we included several questions/prompts that asked people to tell us in their own words, drawing on their direct experience, about fundamental care that had gone well or had not gone well and their view as to the reasons for its success or failure. We were able to explore how views differed between groups, whilst also identifying common ground-the issues that were seen as priorities for patients/public and staff alike.

We needed to involve the research team in the process, as ultimately the research will be undertaken by them, but to find a way of doing so without allowing the researchers’ perspective to dominate. The ‘voice’ of the researcher/academic can be hard to identify in many priority setting tasks. Recognising that they have ample opportunity to shape what is on the table in terms of topics/questions to be prioritised, and that they will be involved in ‘backroom’ capacity in processing responses and identifying potential areas for research, they are typically not included in a priority setting exercise. The intention is to give more voice to the views of patient/public and clinicians who might otherwise not have opportunity to influence which research is prioritised. Yet, especially in our case where it is the research team who is seeking to establish priorities, rather than an external funder, the views of the researchers will have some influence. Our goal was not to eliminate this influence-as the researchers have something valuable to offer to understand the current field of research-but rather to make that contribution explicit and set boundaries to contain it, so that it did not dominate.

The involvement of the Patient Leader-Service User at every stage was the key to this-from the first conversations about the design of the approach, to conducting the consultation and workshop, and through to applying the priorities to the design of a new study. This was exemplified in the decision about whether the researchers at the workshop should be given a chance to ‘vote’ for the top priorities; the final decision was that they should not. The Patient Leader-Service User within the core team also helped ensure that the ‘researcher perspective’ did not dominate, either overtly or covertly.

We have reached an understanding of fundamental care and the research that can improve it, that would not have been possible without the involvement and engagement of a wide range of people, and without assiduous attention to involvement of patients, carers, members of the public, and clinicians throughout the process. We started with a broad perspective-anything was possible-and arrived at a list of priorities that we are using to shape NIHR CLAHRC Wessex applied research into fundamental care.

Our next step has been to identify knowledge gaps related to the priorities and build on the relationships that the priority setting exercise has created to jointly design research that can address the priorities. For example, much research has been done on nurse staffing levels. A review of the literature shows that there is a well-established association between nurse staffing levels and the quality and outcomes of care in hospitals. But a recent review suggests that whilst a substantial volume of work has been undertaken, relatively little of it can be applied in practice [27]. This is an area of research we are now taking forward.

In responding to the priorities identified we are also working with stakeholders and partners to devise a set of interventions that enable staff to involve and communicate with patients to more fully understand individual patient needs and to undertake effective care activities to meet them, tailoring known best practice to suit the needs of the individual, with the outcomes of care assessed and reviewed by the staff providing that care.

Although our research can only address a small number of the issues surfaced and priorities identified, by sharing these priorities, we aspire to inform research and implementation that aims to improve fundamental care in hospital wards far beyond Wessex-nationally and internationally.

## Conclusion

The priority issues for research to improve fundamental are: nurse staffing, individualised/patient-centred care, involvement in care, communication, and staff attitudes.

The inclusion of a Patient Leader-Service User in the core team changed how the research team operated, encouraging sharing and more explicit decision making. It helped to make the project design more open, flexible and inclusive.

Involving a wide range of stakeholders in identifying and prioritising issues in the delivery of care has elicited a complex picture of the scope of fundamental care, with many linked elements. The process we followed has allowed for a different way of conceptualising fundamental care and research needs. It permitted full expression of differences, but provided a means of reaching consensus as to what the important issues are that future research needs to focus on, to improve fundamental care on hospital wards. The process of involvement and engagement led us to a new perspective on fundamental care-what it is, and the factors that enable it.

Benefits go beyond the priority setting as a single discrete task. We have established a connection and form of engagement with members of the public, patients, carers, and health service staff which we will foster, thus enriching the programme of research stemming from their involvement, which we will be working on over the coming years.

## Abbreviations

PPI: Patient and public involvement
